# Divergent adaptations to salinity fluctuations in mangroves: opportunistic photosynthetic leveraging vs. conservative stress resistance in *Aegiceras corniculatum* and *Bruguiera gymnorhiza*

**DOI:** 10.3389/fpls.2026.1877096

**Published:** 2026-07-13

**Authors:** Li Xu, Rui-Li Li, Qian-Quang Liu, Wen-Qing Wang

**Affiliations:** 1 Key Laboratory of Tropical Marine Ecosystem and Bioresource, Fourth Institute of Oceanography, Ministry of Natural Resources, Beihai, China; 2 Key Laboratory of the Coastal and Wetland Ecosystems (Xiamen University), Ministry of Education, College of the Environment and Ecology, Xiamen University, Xiamen, China; 3 Guangxi Key Laboratory of Beibu Gulf Marine Resources, Environment and Sustainable Development, Fourth Institute of Oceanography, Ministry of Natural Resources, Beihai, China; 4 School of Environment and Energy, Shenzhen Graduate School of Peking University, Shenzhen, China

**Keywords:** mangrove plant, osmotic regulation, photosynthesis, salinity fluctuation, stomatal conductance

## Abstract

Large-scale mangrove mortality associated with salinity fluctuations is frequently reported. These fluctuations are caused by climatic extremes or hydraulic engineering projects. However, the adaptation processes of mangrove plants to salinity fluctuations remain poorly understood. We subjected the salt-secreting species *Aegiceras corniculatum* and the salt-excluding species *Bruguiera gymnorhiza* to daily salinity fluctuations, and investigated their responses in terms of gas exchange, PSII photochemical efficiency, and osmotic regulation. *A. corniculatum* demonstrated an opportunistic strategy by efficiently utilizing optimal salinity conditions (5–15‰) during early salinity fluctuations (Day 1). It rapidly modulated stomatal conductance to enhance photosynthetic rate. At the same time, it employed inorganic ion mediated osmotic adjustment to maintain water uptake capacity and sustain stomatal opening. Notably, even when photosynthetic activity was suppressed under high salinity stress (25‰), this species successfully exploited subsequent low salinity periods (5‰) to fully restore photosynthetic performance within a defined recovery period (by Day 35). In contrast, *B*. *gymnorhiza* adopted a conservative approach. It responded to rising salinity by promptly reducing both stomatal conductance and photochemical efficiency to prevent excessive water loss, although this occurred at the expense of photosynthetic rate. *B*. *gymnorhiza* maintained stable leaf inorganic ion concentrations and osmotic potential. This strategy effectively avoided ion toxicity but resulted in reduced water absorption capacity. Consequently, photosynthetic inhibition persisted during transient low salinity intervals. These findings highlight that opportunistic species thrive in dynamic estuarine environments through energy efficient adaptations involving stomatal regulation and inorganic ion homeostasis. In contrast, conservative strategies, while protective against stress, demonstrated limited resilience to salinity fluctuation regimes.

## 1 Introduction

Salinity is a critical factor that limits water availability for plants, particularly in mangrove ecosystems (Ball, 1988; McDowell et al., 2008; Panta et al., 2018). Mangrove plants are located in the intertidal zone. They experience significant fluctuations in topsoil salinity caused by tidal inundation, rainfall, and extreme climatic events (He et al., 2014; Lovelock et al., 2017; Wang et al., 2024). Water salinity in most estuaries exhibits marked seasonal and daily fluctuations. For example, such fluctuations occur in the Huangzhu River, a subtropical river that enters the sea in China (Xu et al., 2023). They also occur in the Saltwater River and Kubaba Rivers in Australia, as well as in the Shark River and Taylor River in southern Florida and other tropical regions (Benfer et al., 2007; Childers et al., 2006; Drushka et al., 2014). In tropical Pacific regions, extreme weather events such as storms, typhoons, and El Niño occur frequently. Mangrove plants in these regions must cope with highly variable topsoil salinity levels ranging from freshwater to seawater (Elster, 2000). In general, topsoil salinity in mangrove habitats is characterized by seasonal, daily, and transient fluctuations (Jaramillo et al., 2018; Naidoo, 1989; Xu et al., 2020).

Large-scale mangrove mortality associated with salinity fluctuations has been widely reported. These fluctuations are caused by climatic extremes or hydraulic engineering projects. The increasing frequency and intensity of cyclones, climatic extremes, and sea level variability in recent years may directly influence mangrove mortality and recovery (Sippo et al., 2018). For example, the 2015 El Niño-Southern Oscillation (ENSO) event caused a sea level decrease in Mangrove Bay, north-west Australia. It also caused a 20–30% rise in soil salinity above pre event levels. These changes led to the loss of 40 hm^2^ of mangrove forest canopy (Lovelock et al., 2017). Similarly, a dieback event in the Gulf of Carpentaria, Australia, affected over 7,400 hm^2^ of mangrove plants (Duke et al., 2017). The construction of hydraulic projects can also rapidly alter the water salinity regime by controlling freshwater inflow. In India, the Farakka Dam reduced freshwater inputs from the Ganges River. This led to the disappearance of the salt sensitive species *Heritiera littoralis*, while the salt tolerant *Avicennia marina* flourished. In contrast, the Koyana Dam increased freshwater flow in the Vashisti River. This resulted in a nearly freshwater regime over approximately 32 km downstream. Consequently, large areas of mangrove forest along the river disappeared. These mangrove degradation and mortality events are all related to salinity fluctuations. However, the underlying mechanisms have not yet been fully demonstrated. Although plant salt tolerance mechanisms have been intensively studied (Chi et al., 2024; Jiang et al., 2026; Naidoo et al., 2011; Thomas et al., 2023), traditional studies of plant adaptation to salinity have predominantly focused on high salinity or steady state salinity. They have paid limited attention to salinity fluctuations.

Recent research on mangrove plant adaptation to salinity fluctuations has focused primarily on gas exchange responses. For example, Ball and Farquhar (1984) increased salinity stepwise from 3‰ to 29‰ and then back to 3‰. They found that the photosynthesis rate (*P*_n_) and stomatal conductance (*G*_s_) of *A. marina* seedlings decreased at salinities above 15‰. However, both parameters recovered substantially when salinity returned to lower levels. In another study, Reef et al. (2015) rapidly transferred the root systems of *A. marina* from a salinity of 35‰ to a high salinity of 65‰ for half an hour. Under this condition, *P*_n_ decreased significantly. In contrast, when root systems were rapidly transferred from 35‰ to a low salinity of 5‰, water uptake increased and *G*_s_ increased significantly. Notably, *P*_n_ and *G*_s_ of mangrove plants can be highly responsive to salinity fluctuations.

Moreover, there is interspecific variation in mangrove species respond to salinity fluctuations. Bompy et al. (2014) exposed *Rhizophora mangle* and *A. germinans* to 40‰ salinity stress for five weeks. This exposure was followed by two weeks of freshwater supplementation. Under these conditions, the mortality rates of these two species did not decrease. In contrast, the salt sensitive species *Laguncularia racemosa* showed significant increases in both *P*_n_ and biomass after freshwater supplementation. However, current research still lacks a systematic understanding of the regulatory mechanisms that control photosynthetic rate under salinity fluctuation conditions. This gap is especially in studies on the adaptation mechanisms of mangrove plants to daily salinity fluctuations driven by tidal cycles.

We conducted a laboratory experiment to determine how mangrove plants respond to daily salinity fluctuations. Previous findings from this experiment demonstrated that salinity fluctuations with prolonged freshwater exposure and a brief high salinity episode increase leaf photosynthetic rate. They also increase the capacity to regulate photoelectron uptake and transfer, as well as ion concentiations (Na^+^, Cl^-^, K^+^) (Wang et al., 2020). In the present study, we simulated daily salinity fluctuations. Building on previous findings, we investigated the effects on these fluctuations on two mangrove species: the salt secreting *Aegiceras corniculatum* and the salt excluding *Bruguiera gymnorhiza*. We also explored the differences between the two species in their responses to salinity fluctuations. Understanding these differences is crucial for elucidating the mechanisms of adaptation to salinity fluctuations. It is also important for understanding the distribution patterns of mangrove forests in estuaries. We propose the following hypothesis: the adaptive strategies of the two species to salinity fluctuations will differ. Species that employ energy efficient strategies involving rapid stomatal conductance modulation and ion-mediated osmotic adjustment are likely better adapted to salinity fluctuations. Such species may thrive in estuarine zones.

## 2 Material and methods

### 2.1 Seedling culture

*A. corniculatum* and *B. gymnorhiza* are among the most common mangrove plants in tropical and subtropical regions. Both species exhibit moderate salt tolerance. They struggle to thrive at salinities of 30‰ (Ball, 1988; Burchett et al., 1989; Takemura et al., 2000). Mature propagules of these species were collected from the Futian Mangrove Nature Reserve in China (22°32′N, 114°03′E) in September 2017. The propagules were of similar size. Their heights were 3.5 ± 0.3 cm and 18.5 ± 1.6 cm, respectively. Their fresh weights were 2.2 ± 0.2 g and 22.1 ± 1.7 g, respectively. The propagules were initially cultivated at Peking University Shenzhen Graduate School in Shenzen, China. Each propagule was planted in a 0.5 L plastic pot filled with sand. The pots were irrigated with tap water until the water level reached the sand surface. The plants were grown under a rain shelter. The maximum photosynthetically active radiation (PAR) at midday was 750 µmol m^-2^s^-1^. During the cultivation period, all seedlings were fertilized twice. Fertilization occurred after 5 weeks and after 20 weeks. A complex fertilizer was used (N:P:K = 1:1:1; manufactured by JSC ACRON, Russia). After approximately 20 weeks (March 2018), the healthiest seedling were transferred to opaque plastic containers (35 cm × 25 cm × 18 cm). Six seedlings were planted per container. They were grown in 20% Hoagland’s nutrient solution (pH 6.8). Their root systems were fully submerged. The nutrient solution was replaced weekly. All seedlings were grown in an environmentally controlled growth cabinet (Thermoline^®^ Scientific Equipment, TPG-6000-TH-CO_2_, Smithfield, NSW, Australia). Environment conditions were maintained as follows: temperature at 25.0–27.0 °C, relative humidity at 70%–75%, CO_2_ concentration at 450–500 ppm, and a 13 hour photoperiod (8:20–21:20). The PAR at leaf height was maintained at 450–500 µmol m^-2^s^-1^.

### 2.2 Salinity treatments

On 25 March 2018, 84 seedlings of each species were selected. All selected seedlings were of similar size. They were simultaneously assigned to two salinity treatments. Each treatment had six replicates and 42 seedlings per treatment. At the time of treatment application, seedling height ranged from 6.9 to 8.5 cm for *A. corniculatum* and from 33.3 to 40.9 cm for *B. gymnorhiza.* The seedlings had 2–3 pairs of leaves.

Salinity was applied to the entire root system. The two treatments were daily salinity fluctuation (F) and steady state salinity (C; control). A salinity of 29‰ is known to induce growth stress in both species (Ball, 1988; Zhu et al., 2012). The F treatment was designed based on salinity variations observed in the surface soil layer of subtropical estuaries (Benfer et al., 2007; Xu et al., 2020). Specifically, salinity was increased daily from 5‰ to 15‰, then to 25‰, and subsequently decreased back to 15‰ and then to 5‰. Each salinity level was maintained for three hours, and one complete salinity cycle was completed per day. In contrast, the control was maintained at 5‰ throughout the experiment. The control salinity was set to 5‰ because this value is near the optimal salinity for seedlings of both species. Additionally, it represents a low salinity level that can alleviate high salt stress under salinity fluctuations (Wang et al., 2011). Each salinity treatment lasted 35 days. Salinity levels were adjusted using a combination of 20% Hoagland’s nutrient solution and NaCl. The culture solutions for all treatments were replaced weekly.

**Figure 1:**
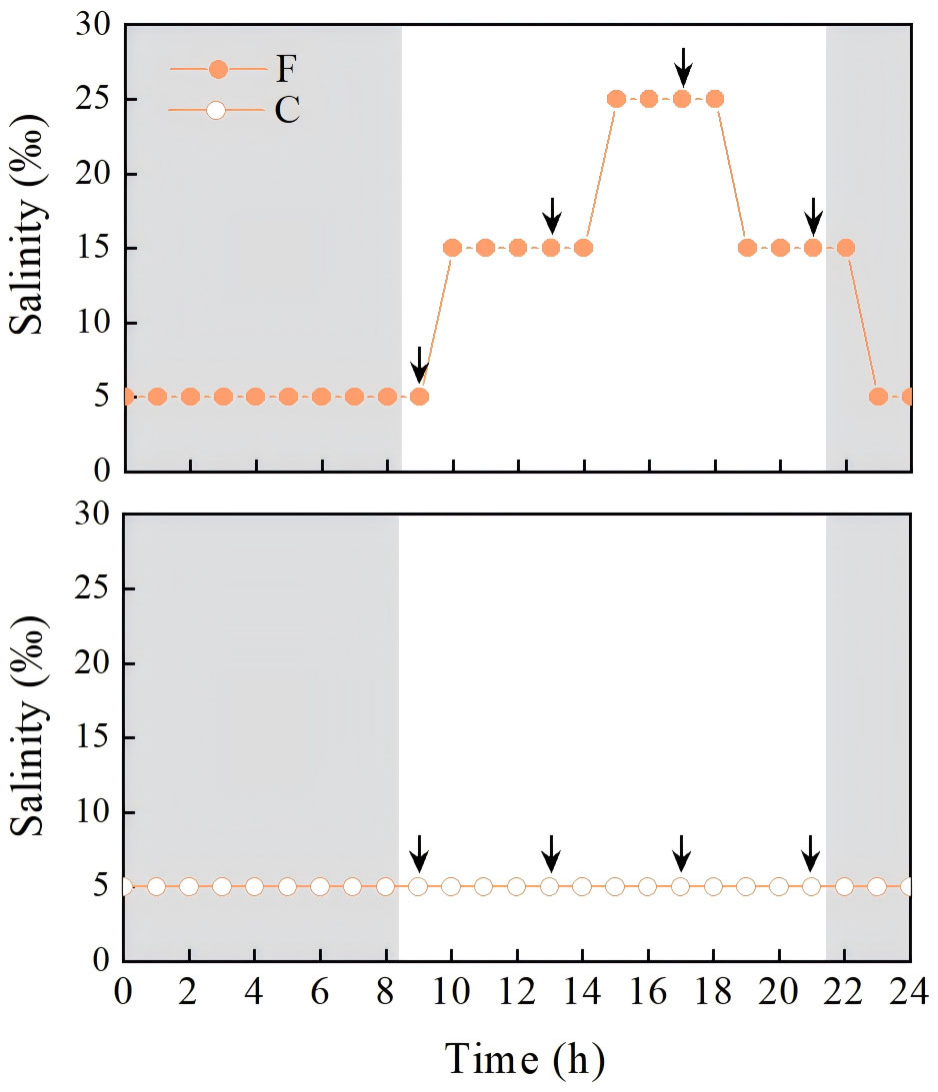
Salinity management over time for different salinity regimes. For daily salinity fluctuation (F), salinity in the root zone was subjected to stepwise changes every day. It increased from 5‰ to 15‰, then to 25‰, and subsequently decreasing back to 15‰ and then to 5‰. For the constant salinity treatment (C; control), salinity in the root zone was maintained at a constant 5‰ throughout the experiment. Arrows indicate the time points at which measurements of leaf gas exchange, chlorophyll fluorescence, ion concentration, moisture content and osmotic potential were taken. The gray patch indicates the dark period in the growth chamber (21:20 to 08:20).

Measurements of leaf gas exchange, chlorophyll fluorescence, ion concentration, moisture content, and osmotic potential were taken at four daily time points (09:00, 13:00, 17:00, and 21:00). These measurements were conducted on the 1st, 7th, 14th, and 35th days of the salinity treatments. These time points corresponded to varying salinity levels in the F treatment, specifically 5‰, 15‰, 25‰, and 15‰, respectively (**Figure 1**).

### 2.3 Measurement of leaf gas exchange parameters

A Li-6400 portable photosynthesis system (Li-Cor, Lincoln, Nebraska, USA) was used. Leaf gas exchange parameters were measured on the newest fully expanded leaf of six replicate seedlings from each treatment. The leaf chamber had an area of 6.25 cm^2^. To reduce measurement errors, the measurement conditions were maintained as consistent as possible with the environment inside the growth chamber. The measurement conditions were as follows: photosynthetically active radiation (PAR) of 500 µmol m^−2^s^−1^ on the leaf surface, leaf temperature 26.0 °C, ambient CO_2_ concentration of 450 ppm, and water vapor pressure of 1.12–1.22 kPa in the leaf chamber. For each leaf, five readings were recorded. Their means were used to calculate stomatal conductance (*G*_s_), net photosynthesis rate (*P*_n_), transpiration rate (*T*_r_), and intercellular CO_2_ concentration (*C*_i_).

### 2.4 Measurement of chlorophyll fluorescence parameters

Chlorophyll fluorescence measurements were taken on the same leaves. A portable fluorescence system (PAM 2500, Walz, Germany) equipped with a leaf-clip holder (2030-B, Walz) was used. Following gas exchange measurements, the fluorescence yield for instantaneous fluorescence (*F*_s_), maximum fluorescence (*F*_m_′), and minimal fluorescence in the light-adapted state (*F*_o_′) were determined. The protocol of Yuan et al. (2012) was followed. The photochemical efficiency of open PSII (*F*_v_′/*F*_m_′) was calculated using the following formula:

(1) $$F_{m}^{'}/F_{v}^{'}=(F_{m}^{'}-F_{o}^{'})/F_{m}^{'}$$

The efficiency of Photosystem II (*Φ*_PSII_) and the non-photochemical quenching coefficient (*NPQ*) were calculated using the following formulas (Kato et al., 2003; Kramer et al., 2004):

(2) $${\text{Φ}}_{\text{PSII}}=(F_{m}^{'}-\;F_{s})/F_{m}^{'}$$

(3) $$NPQ=F_{s}/F_{m}^{'}-1$$

### 2.5 Measurement of moisture content and ions

The seedlings were sampled and thoroughly rinsed with deionized water to remove surface salts. Roots, stems, propagules, and leaves were separated. Their wet and dry weights were measured after oven-dried at 75 °C for 72 hours. Inorganic ions (Cl^–^, Na^+^ and K^+^) were quantified using inductively coupled plasma optical emission spectrometry (IC-OES; Optima 7000 DV, Perkin-Elmer, USA), following the protocol described by Genc et al. (2010). Moisture content (MC) was calculated using the following formula:

(4) MC=(FW−DW)/DW

where MC is moisture content, FW is fresh weight, and DW is dry weight.

### 2.6 Measurement of osmotic potential

The leaves were harvested and immediately frozen in liquid nitrogen to preserve their physiological state. After a brief freezing period, the leaves were thawed at room temperature. The extracted sap was then collected to measure osmotic potential (*Ψ*_s_) using a vapor pressure osmometer (Wescor 5520, Logan, USA).

### 2.7 Statistical analysis

Statistical differences among time points and treatments were evaluated using analysis of variance (ANOVA) in SPSS v. 18.0 (Chicago, IL, USA). Duncan’s test was used for mean separation. Differences in physiological parameters across salinity gradients and treatments were analyzed using one-way ANOVA. All graphs were generated using R (version 4.3.0).

## 3 Results

### 3.1 Rapid responses of gas exchange parameters in *A. corniculatum* and *B. gymnorhiza* to salinity fluctuations

**Figure 2:**
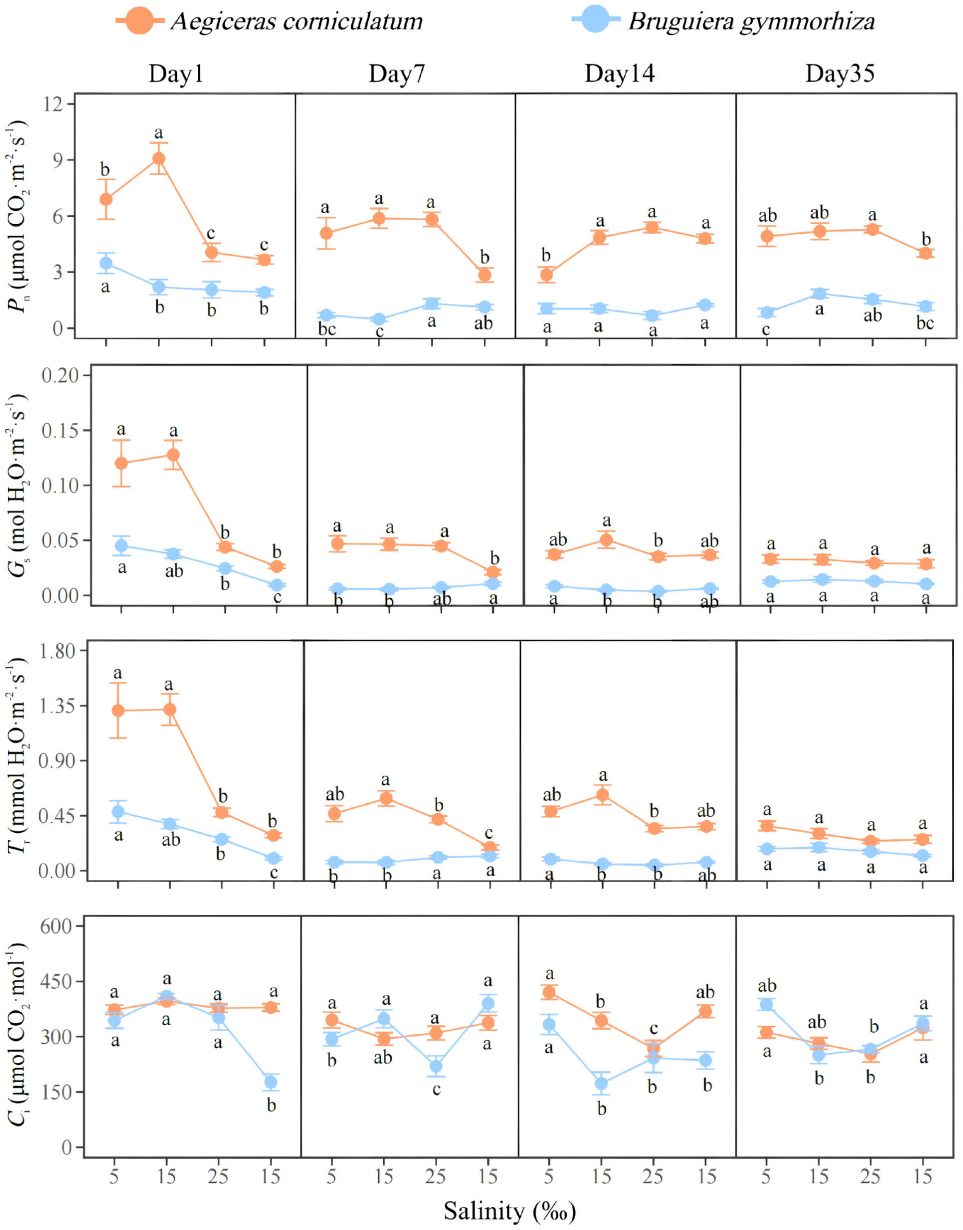
Responses of leaf gas exchange of *Aegiceras corniculatum* and *Bruguiera gymnorhiza* seedlings to daily fluctuating salinity. Data are means ± SE (*N* = 5–6). Different lowercase letters above bars indicate significant differences among different salinity, according to Duncan’s test at *P* < 0.05. *G*_s_ stomatal conductance; *P*_n_ net photosynthesis rate; *T*_r_ transpiration rate; and *C*_i_ intercellular CO_2_ concentration.

During the initial phase of salinity fluctuations (Day 1), leaf gas exchange parameters of *A. corniculatum* and *B. gymnorhiza* seedlings exhibited a rapid response to the salinity increase. In *A. corniculatum*, increasing salinity from 5‰ to 15‰ enhanced *P*_n_, *T*_r_, *G*_s_, and *C*_i_. However, a further increases to 25‰ suppressed these parameters. In contrast, *B. gymnorhiza* exhibited immediate inhibition of gas exchange even under a mild salinity rise (5–15‰). Notably, reducing salinity from 25‰ to 15‰ did not mitigate the suppressive effects in either species (**Figure 2**).

**Figure 3:**
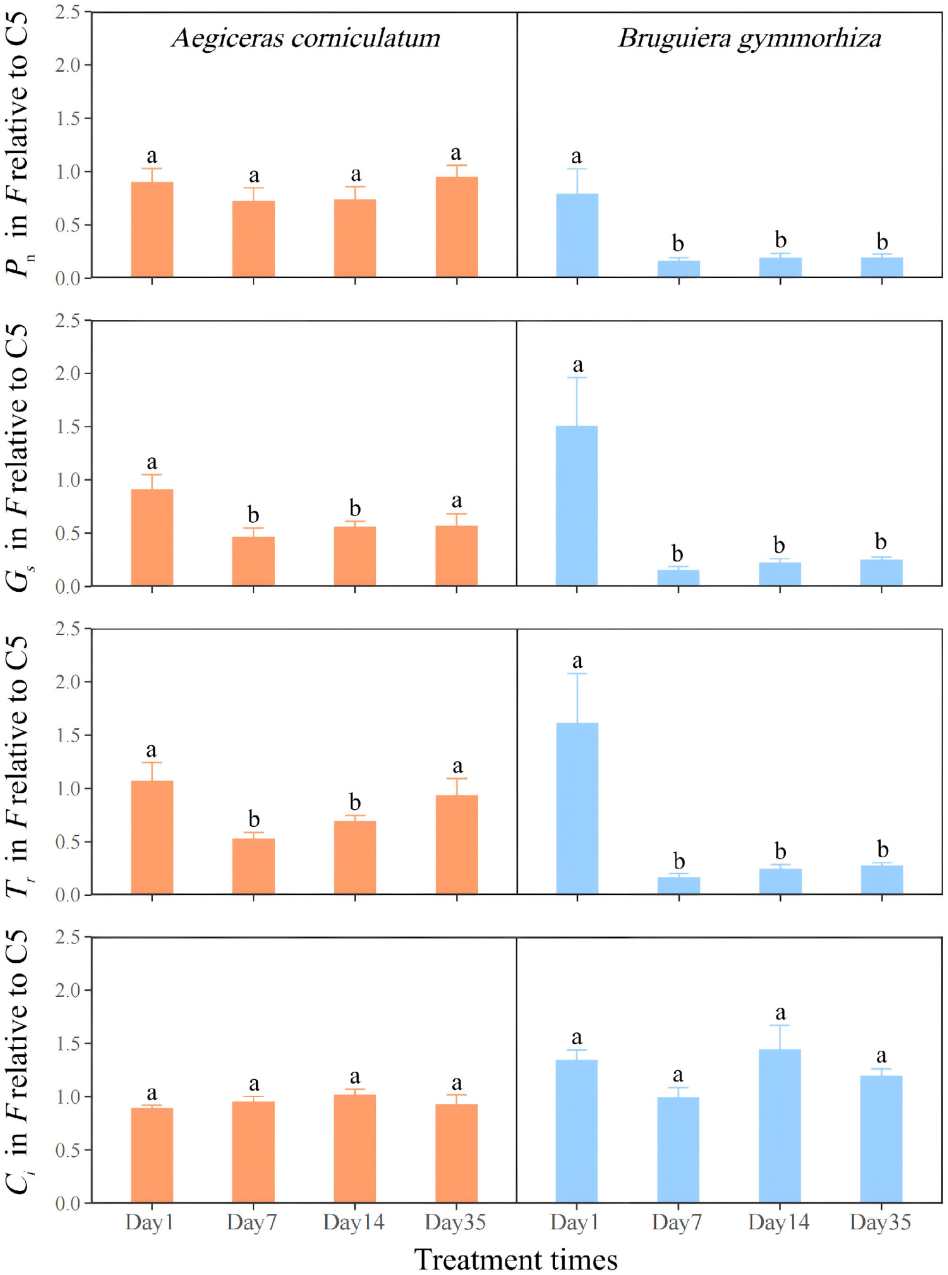
Gas exchange of *Aegiceras corniculatum* and *Bruguiera gymnorhiza* seedlings as a proportion of the daily fluctuating salinity treatment relative to the control. Data are means ± SE (*N* = 5–6). Different lowercase letters above bars indicate significant differences among different treatment times, according to Duncan’s test at *P* < 0.05. Day 1, 7, 14, and 35 refer to the first day, seventh, the 14th, and 35th days of the salinity treatment, respectively. *G*_s_ stomatal conductance; *P*_n_ net photosynthesis rate; *T*_r_ transpiration rate; and *C*_i_ intercellular CO_2_ concentration.

Compared to the steady state salinity (5‰; control), *A. corniculatum* exhibited recover in *P*_n_, *T*_r_ and *G*_s_ on Day 35. In contrast, *B. gymnorhiza* maintained reduced levels after an initial drop on Day 1. These results demonstrate that low-salinity phases mitigated the photosynthetic inhibition caused by high salinity in *A. corniculatum.* However, they remained ineffective against high salinity induced photosynthetic suppression in *B. gymnorhiza* (**Figure 3**).

### 3.2 Rapid responses of photochemical efficiency in *A. corniculatum* and *B. gymnorhiza* to salinity fluctuations

**Figure 4:**
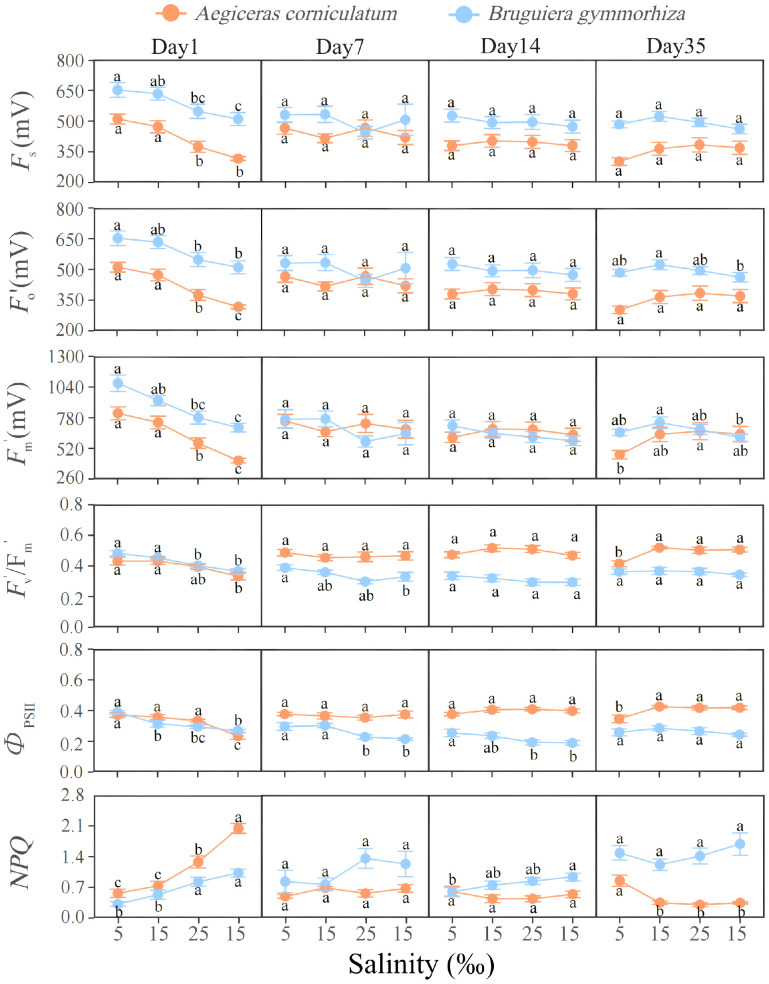
Responses of chlorophyll fluorescence of *Aegiceras corniculatum* and *Bruguiera gymnorhiza* seedlings to daily fluctuating salinity. Data are means ± SE (*N* = 5–6). Different lowercase letters above bars indicate significant differences among different salinity, according to Duncan’s test at *P* < 0.05. *F*_s_ the fluorescence yield for instantaneous fluorescence; *F*_m_′ maximum fluorescence, *F*_o_′ minimal fluorescence in the light-adapted state; *F*_v_′/*F*_m_′ the photochemical efficiency of open PSII; *Φ*_PSII_ the efficiency of Photosystem II; *NPQ* the non-photochemical quenching coefficient.

Fluctuating hypersalinity significantly inhibited photochemical efficiency and increased heat dissipation in both *A. corniculatum* and *B. gymnorhiza.* Specifically, *F*_s_, *F*_o_′, and *F*_m_′ decreased significantly when salinity initially increased to 25 ‰ (Day 1). Consequently, *F*_v_′/*F*_m_′ (Equation 1) and *Φ*_PSII_ (Equation 2) were reduced, while NPQ (Equation 3) increased (**Figure 4**).

**Figure 5:**
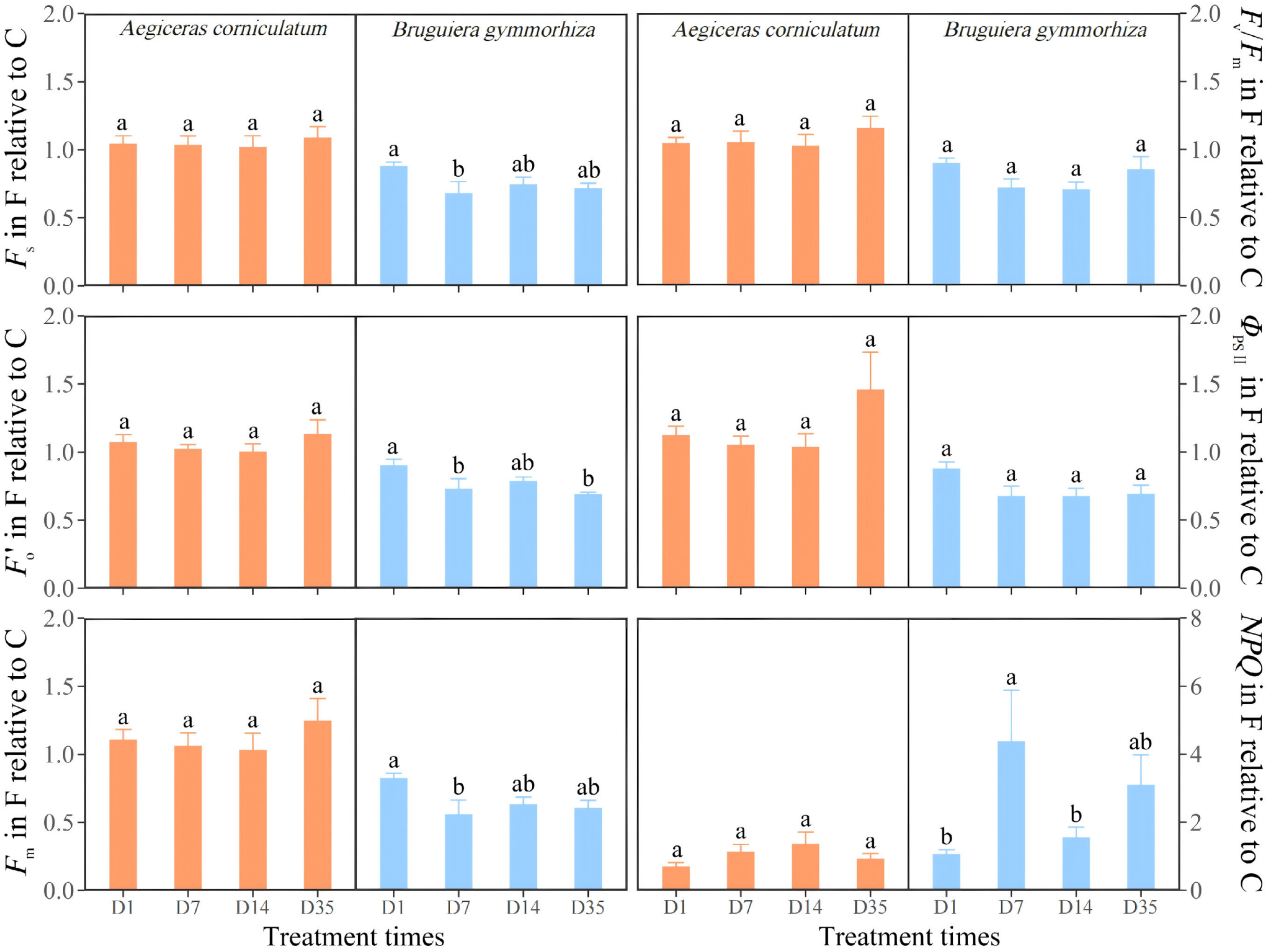
Chlorophyll fluorescence of *Aegiceras corniculatum* and *Bruguiera gymnorhiza* seedlings as a proportion of the daily fluctuating salinity treatment relative to the control. Data are means ± SE (*N* = 5–6). Different lowercase letters above bars indicate significant differences among different treatment times, according to Duncan’s test at *P* < 0.05. *F*_s_ the fluorescence yield for instantaneous fluorescence; *F*_m_′ maximum fluorescence, *F*_o_′ minimal fluorescence in the light-adapted state; *F*_v_′/*F*_m_′ the photochemical efficiency of open PSII; *Φ*_PSII_ the efficiency of Photosystem II; *NPQ* the non-photochemical quenching coefficient.

During the later phase (Day 35), the photochemical efficiency parameters (*F*_s_, *F*_o_′, *F*_m_′, *F*_v_′/*F*_m_′, *Φ*_PSII_ and NPQ) in *A. corniculatum* seedlings were comparable to those of the control seedlings. This finding demonstrates the buffering effect of low salinity against high hypersalinity. In contrast, *B. gymnorhiza* did not recover its photochemical efficiency (**Figure 5**).

### 3.3 Salinity fluctuations reduced moisture content in leaves and whole seedlings of both species

Salinity fluctuations significantly impacted the moisture content (Equation 1) of leaves and whole seedlings in both *A. corniculatum* and *B. gymnorhiza*. In *A. corniculatum*, the moisture content of whole seedling, stem and propagule increased on Day 7 but decreased on Day 35. In *B. gymnorhiza*, the moisture content of whole seedlings, leaves, and propagules consistently decreased under salinity fluctuations. Notably, *A. corniculatum* exhibited significantly higher moisture content in whole seedlings compared to *B. gymnorhiza* during the later phase (Day 35) (*P* < 0.05; **Table 1**).

**Table 1:** Responses of moisture content of *Aegiceras corniculatum* and *Bruguiera gymnorhiza* seedlings to daily fluctuating salinity.

Species	Time	Leaf	Stem	Hypocotyl	Root	Whole seedling
*A. corniculatum*	Day 1	2.42 ± 0.033^a^	2.20 ± 0.15^a^	2.30 ± 0.14^b^	3.46 ± 0.09^b^	2.65 ± 0.11^b^
	Day 7	2.16 ± 0.11^ab^	2.24 ± 0.34^a^	2.88 ± 0.23^a^	4.80 ± 0.20^a^	3.06 ± 0.18^a^
	Day 35	2.01 ± 0.12^b^	1.38 ± 0.06^b^	1.69 ± 0.05^c^	3.26 ± 0.09^b^	2.22 ± 0.05^c^
*B. gymnorhiza*	Day 1	2.36 ± 0.09^a^	1.95 ± 0.05^a^	1.78 ± 0.04^a^	2.93 ± 0.15^a^	2.05 ± 0.04^a^
	Day 7	2.27 ± 0.10^a^	1.97 ± 0.07^a^	1.67 ± 0.07^ab^	2.94 ± 0.10^a^	1.93 ± 0.08^ab^
	Day 35	1.91 ± 0.15^b^	1.81 ± 0.09^a^	1.55 ± 0.01^b^	2.76 ± 0.19^a^	1.80 ± 0.02^b^

Data are means ± SE (*N* = 12). Each data is the mean value of four time point (09:00, 13:00, 17:00, and 21:00), and there are three replication at each time point. Different lowercase letters indicate significant differences among different salinity times, according to Duncan’s test at *P* < 0.05.

### 3.4 Contrasting effects of salinity fluctuations on osmotic regulation by inorganic ions in two species

**Figure 6:**
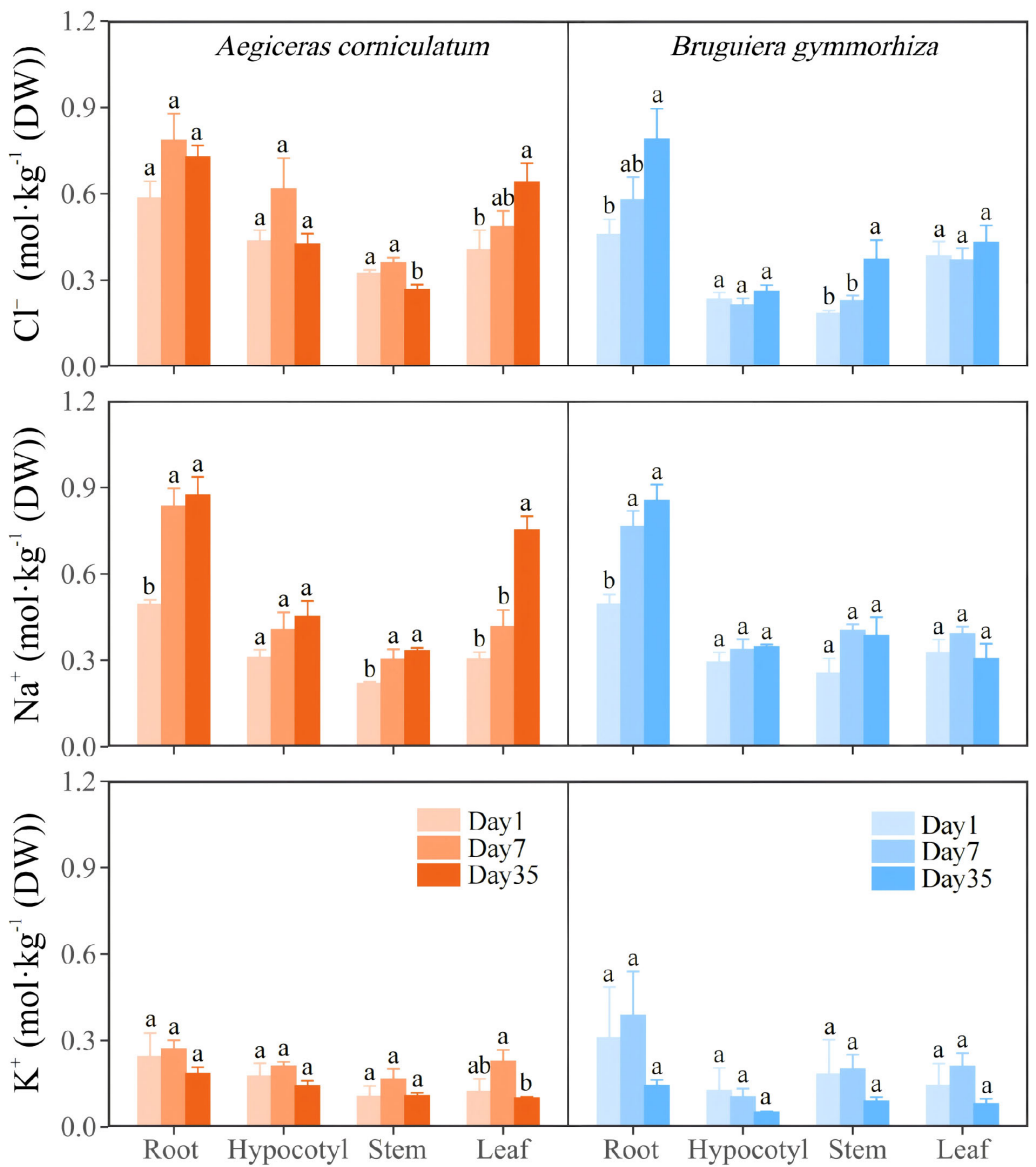
Responses of inorganic ion concentration of *Aegiceras corniculatum* and *Bruguiera gymnorhiza* seedlings to daily fluctuating salinity. Data are means ± SE (*N* = 12). Each data is the mean value of four time point (09:00, 13:00, 17:00, and 21:00). Different lowercase letters above bars indicate significant differences among different treatment times for the same seedling organ, according to Duncan’s test at *P* < 0.05.

Salinity fluctuations exhibited contrasting effects on osmotic regulation mediated by inorganic ions between the two species. In *A. corniculatum*, salinity fluctuations significantly increased Cl^-^ and Na^+^ contents in both roots and leaves (*P* < 0.05). In contrast, salinity fluctuations in *B. gymnorhiza* had no significant effect on Cl^-^ and Na^+^ contents in the leaves (*P >* 0.05, **Figure 6**).

**Figure 7:**
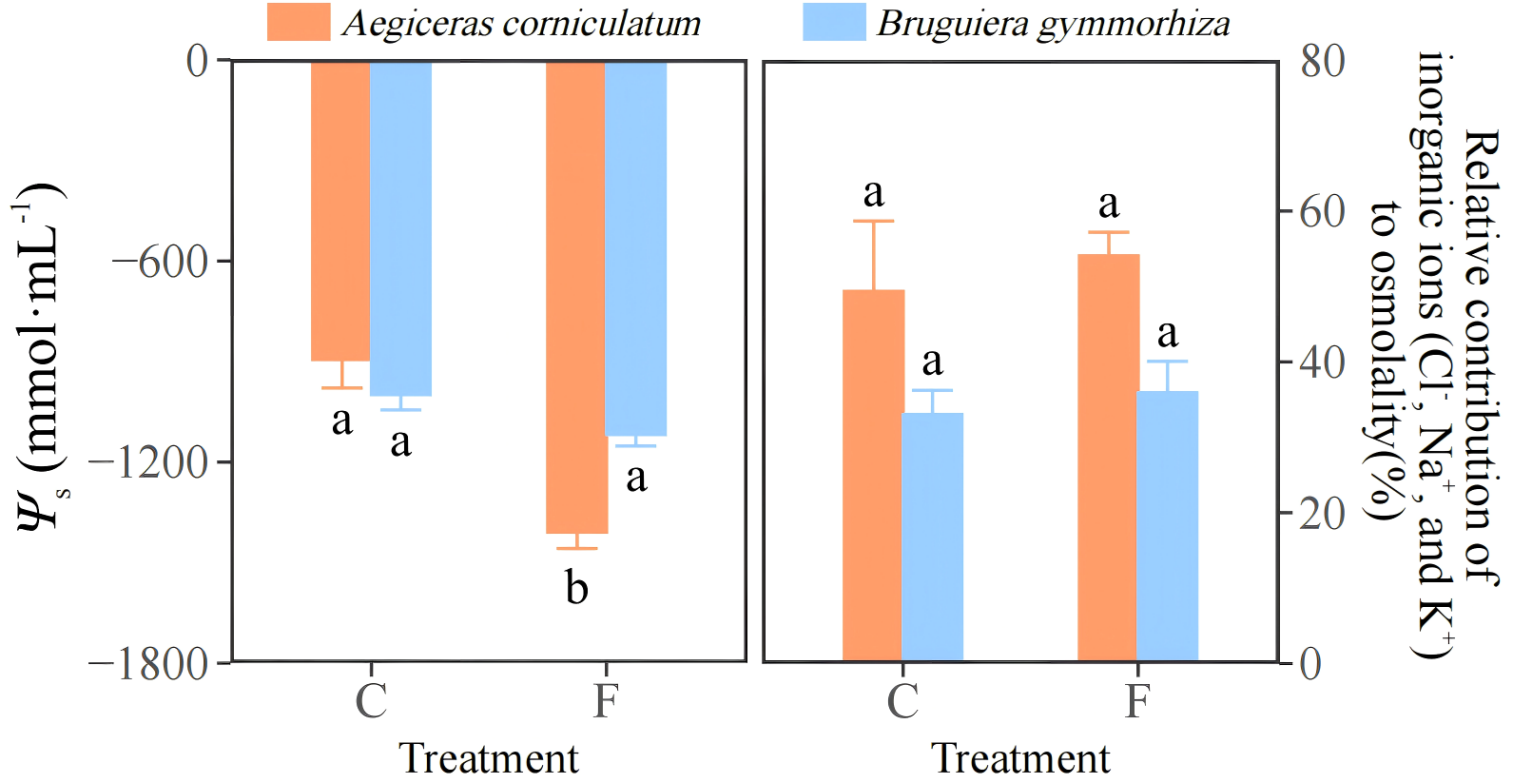
Responses of osmotic potential of *Aegiceras corniculatum* and *Bruguiera gymnorhiza* seedlings to daily fluctuating salinity. Data are means ± SE (*N* = 12). Each data is the mean value of four time point (09:00, 13:00, 17:00, and 21:00), and there are three replication at each time point. Different lowercase letters above bars indicate significant differences among different salinity treatments, according to Duncan’s test at *P* < 0.05.

Salinity fluctuations increased leaf *Ψ*_s_ in *A. corniculatum* but had no significant effect on leaf *Ψ*_s_ in *B. gymnorhiza*. In both species, the contribution of inorganic ions (Cl^-^, Na^+^ and K^+^) to leaf *Ψ*_s_ was similar to that of the control. However, the contribution of inorganic ions to leaf *Ψ*_s_ was significantly higher in *A. corniculatum* than in *B. gymnorhiza*. Specifically, the contribution ratios were 54.05% for *A. corniculatum* and 35.91% for *B. gymnorhiza* (**Figure 7**).

## 4 Discussion

### 4.1 Contrasting adaptive strategies of opportunistic and conservative mangrove species in response to salinity fluctuations

Mangrove plants face constrained water availability due to multiple factors. Salinity emerges as a particularly critical stressor in these ecosystems (McDowell et al., 2008; Panta et al., 2018). While traditional photosynthesis research has emphasized steady state conditions, mangrove habitats actually experience frequent salinity fluctuations. Most species demonstrate specific optima for growth (8.8‰ for *A. corniculatum* and 7.3‰ for *B. gymnorhiza*) (Wang et al., 2011). However, they generally show reduced viability when salinity exceeds 30‰ (Mendez-Alonzo et al., 2016; Naidoo et al., 2011). Importantly, the adjustments of CO_2_ assimilation rates during salinity fluctuations often require prolonged periods. This is especially true during the transition back to lower salinity. These adjustments can span from hours to days (Ball and Farquhar, 1984; Reef and Lovelock, 2014). This delayed recovery highlights how mangroves employ distinct adaptive strategies for dynamic versus stable salinity conditions.

The contrasting physiological responses between opportunistic and conservative mangrove species reveal fundamental principles about plant resilience in variable environments. *A. corniculatum*, is a salt secreting species. It exemplifies an adaptation strategy focused on rapid exploitation of favorable conditions. This strategy involves tightly coordinated hydraulic, stomatal, and osmotic responses. By contrast, *B. gymnorhiza* is a salt excluding species. It adopts a more conservative approach that prioritizes cellular protection at the expense of metabolic flexibility. This results in limited recovery capacity when salinity changes abruptly. These divergent strategies represent evolutionary adaptations to different positions along hydroclimatic variability gradients. They do not simply represent differences in tolerance thresholds.

### 4.2 Stomatal regulation as the primary determinant of photosynthetic responses to salinity fluctuations in mangrove plants

The initial impact of salinity stress manifests as osmotic imbalance. This imbalance causes cellular water deficit, which triggers sequential physiological responses (Munns, 2002). Recent research demonstrates that stomatal regulation constitutes the early response to limit water evaporation and loss (Flexas and Medrano, 2002; Gong et al., 2025). In the present study, *P*_n_ showed synchronized variation patterns with *G*_s_ and *C*_i_ across salinity gradients during the initial phase in both species (**Figure 2**). This finding indicates that changes in photosynthetic rate are mainly regulated by stomata. Stomatal conductance directly affects the diffusion of carbon dioxide into mesophyll cells. It therefore constitutes the main limiting factor of photosynthetic rate (Flexas and Medrano, 2002; von Caemmerer and Farquhar, 2025).

*A. corniculatum* employs a “hydraulic anticipation” strategy even under moderate salinity stress (5–15‰). This strategy enables immediate stomatal opening and increased carbon assimilation (Qie et al., 2024). This contrasts with *B. gymnorhiza*’s “hydraulic risk aversion” approach. In this approach, rapid stomatal closure prevents dehydration but leads to prolonged photosynthetic suppression. This suppression persists even after salinity decreases (Cheng et al., 2025).

### 4.3 Contrasting osmotic regulation strategies between salt-secreting and salt-excluding mangrove species under salinity fluctuations

When NaCl is added to 20% Hoagland nutrient solution, the water potential of the solution decreases. This makes it difficult for seedling roots to absorb water, thereby inducing osmotic stress. In response, the roots accumulate inorganic ions or synthesize organic solutes for osmotic adjustment. The preferential accumulation of inorganic ions (Na^+^, Cl^–^, and/or K^+^) for osmotic regulation in mangrove plants is generally well documented (Munns and Tester, 2008; Xu et al., 2020; Zhu et al., 2011). At the same time, this accumulation creates an ion imbalance: excess Na^+^ interferes with K^+^ transporters and displaces Ca²^+^ from cell wall binding sites, compromising membrane integrity. However, the rapid accumulation of these inorganic ions serves as a crucial early response to salinity stress. Therefore, the impacts of ion imbalance is not obvious over short time scales (Munns, 2002).

Osmotic regulation strategies further differentiate these species’ resilience. *A. corniculatum* transiently accumulates Na^+^ and Cl^–^ in leaf tissues during salinity increases. These ions serve as rapidly mobilizable osmolytes. The species then efficiently secretes excess salts via specialized salt glands within hours (Losso et al., 2025). This allows the species to temporarily maintain low leaf water potentials for water uptake. It also helps avoid long term ion toxicity (Xu et al., 2023). Interestingly, similar patterns appear in other salt secreting species such as *A. officinalis* (Tan et al., 2013). For *A. corniculatum*, most salt is initially excluded at the root surface. Excess ions are then transported for excretion. This effectively creates a temporary “salt storage” mechanism (Xu et al., 2023).

Conversely, *B. gymnorhiza* maintains stable leaf ion concentrations through highly efficient root level salt exclusion (approximately 90% Na^+^ rejection) (Krishnamurthy et al., 2014; Scholander, 1968). However, this strategy comes at the cost of limited capacity for rapid osmotic readjustment when salinity declines (Cheng et al., 2025). Lacking salt glands, this species relies on slower acting organic osmolytes such as proline and glycine betaine. These osmolytes cannot respond promptly to natural salinity fluctuations (Wang et al., 2022).

### 4.4 Divergent PSII photochemical efficiency and recovery patterns underlie contrasting resilience of mangrove species to salinity fluctuations

Photosystem II (PSII) photochemical efficiency provides additional insight into these species’ divergent strategies. Under high salinity (25‰), both species show reduced photosynthetic rates, but they differ markedly in their recovery patterns. *A. corniculatum* quickly restores PSII function during low salinity. It shows efficient NPQ relaxation and reactivation of electron transport components (Qie et al., 2024). *B. gymnorhiza*, however, displays persistent NPQ elevation and reduced *F*_v_/*F*_m_ ratios. These indicators suggest chronic photoinhibition. This inhibition results from impaired chloroplast ion homeostasis and delayed repair mechanisms (Wang et al., 2022). These findings challenge earlier observations. Previous studies showed that freshwater supplementation during salinity fluctuations can enhance *B. gymnorhiza*’s recovery. This suggests that its poor performance in the present study may reflect insufficient low salinity duration (Wang et al., 2020). Together, these results confirm that while salt exclusion works well under stable high salinity, it proves disadvantageous in fluctuating conditions. Fluctuating conditions require rapid damage repair cycles.

### 4.5 Ecological implications of contrasting salinity adaptation strategies for mangrove distribution and restoration under climate change

These physiological differences have profound ecological implications for mangrove distribution and survival under climate change. Opportunistic species such as *A. corniculatum* thrive in environments with frequent, moderate salinity fluctuations. Diurnal tidal zones are examples of such environments. In these settings, rapid hydraulic stomatal osmotic coordination determines carbon gain. Conservative species such as *B. gymnorhiza* persist in more stable environments. These environments are often harsher. In these cases, damage prevention outweighs recovery speed. However, climate driven increases in hydroclimatic variability may increasingly favor opportunistic species. These species possess greater phenotypic plasticity (Fazlioglu et al., 2020). Therefore, in ecological restoration areas where salinity changes frequently, priority should be given to these opportunistic species. Furthermore, the construction of hydraulic projects such as canals and dams should be managed to avoid significant impacts on water salinity. Otherwise, such impacts could alter the structural of mangrove communities.

## 5 Conclusions

This study has revealed fundamental differences in salt secreting and salt excluding mangrove plants respond physiologically to salinity fluctuations. These differences have important implications for their growth and distribution patterns. Our findings demonstrate that frequent salinity fluctuations significantly affect mangrove ecosystems. Conservative species such as *B. gymnorhiza* decline due to their limited response capacity. In contrast, opportunistic species such as *A. corniculatum* expand through efficient stomatal regulation and transient ion accumulation. These traits allow them to capitalize on low salinity periods.

The study establishes that salinity fluctuation resilience is an emergent property. It arises from the integrated function of multiple physiological systems operating across different timescales. These systems include hydraulic conductivity, stomatal regulation, and osmotic adjustment. The contrasting strategies of *A. corniculatum* and *B. gymnorhiza* represent distinct evolutionary solutions. These solutions respond to varying selective pressures along environmental variability gradients. Future research should target direct measurements of root to leaf hydraulic conductivity dynamics. Such measurements would test the hypothesis that photosynthetic recovery following salinity stress depends primarily on restoring water transport capacity (Li et al., 2025). This line of research will prove essential for accurately predicting mangrove ecosystem responses to accelerating environmental changes. It will also help develop effective conservation approaches for these ecologically vital coastal habitats.

## Data Availability

The original contributions presented in the study are included in the article/supplementary material. Further inquiries can be directed to the corresponding author.
