# Correction: Divergent adaptations to salinity fluctuations in mangroves: opportunistic photosynthetic leveraging vs. conservative stress resistance in *Aegiceras corniculatum* and *Bruguiera gymnorhiza*

**DOI:** 10.3389/fpls.2026.1939415

**Published:** 2026-07-23

**Authors:** Frontiers Production Office

**Affiliations:** Frontiers Media SA, Lausanne, Switzerland

**Keywords:** mangrove plant, osmotic regulation, photosynthesis, salinity fluctuation, stomatal conductance

Affiliation “South Valley University, Egypt” was erroneously assigned to Arafat Abdel Hamed Abdel Latef. The correct affiliation is: “Qena University, Egypt”.

The original version of this article has been updated.

